# A Jurassic wood providing insights into the earliest step in *Ginkgo* wood evolution

**DOI:** 10.1038/srep38191

**Published:** 2016-12-16

**Authors:** Zikun Jiang, Yongdong Wang, Marc Philippe, Wu Zhang, Ning Tian, Shaolin Zheng

**Affiliations:** 1 Chinese Academy of Geological Sciences, Beijing 100037, China; 2 State Key Laboratory of Palaeobiology & Stratigraphy, Nanjing Institute of Geology and Palaeontology, Chinese Academy of Sciences, Nanjing 210008, China; 3 UMR 5023 of the CNRS and Université Lyon 1, 7 rue Dubois, F69622 Villeurbanne, France; 4 Shenyang Institute of Geology and Mineral Resources, Shenyang, 110034, China; 5 College of Palaeontology, Shenyang Normal University, Shenyang, 110034, China

## Abstract

The fossil record of *Ginkgo* leaf and reproductive organs has been well dated to the Mid-Jurassic (170 Myr). However, the fossil wood record that can safely be assigned to Ginkgoales has not yet been reported from strata predating the late Early Cretaceous (ca. 100 Myr). Here, we report a new fossil wood from the Mid-Late Jurassic transition deposit (153–165 Myr) of northeastern China. The new fossil wood specimen displays several *Ginkgo* features, including inflated axial parenchyma and intrusive tracheid tips. Because it is only slightly younger than the oldest recorded *Ginkgo* reproductive organs (the Yima Formation, 170 Myr), this fossil wood very probably represents the oldest bona fide fossil *Ginkgo* wood and the missing ancestral form of *Ginkgo* wood evolution.

The maidenhair tree or *Ginkgo* is often described as a “living fossil”. It is one of the very few extant plant genera that can be traced back to the Jurassic, at approximately 170 Myr ago. Since *Ginkgo apodes* Zhou and Zheng^1^, the “missing link” in *Ginkgo* evolution, has been reported from North-Eastern China, the genus has a quite complete fossil record lineage with different organs documented, including leaves, pollen, reproductive structures, and long and dwarf shoots. Such a complete record lineage is of great interest for phylogenetical studies and for deciphering the potential role of developmental heterochronies in the evolution of Ginkgoales^2^.

A tree is made of wood, which forms more than 80% of its biomass. Wood ecophysiology is recognized as a first-order factor in plant evolution^3^. Unfortunately, the fossil wood record for *Ginkgo* is very scanty^4^,^5^, with no established data predating the late Early Cretaceous, i.e., ca. 100 Myr^6^,^7^.

Some woods older than the Cretaceous with supposed and putative relationship links to the Ginkgoales were described as a variety of taxa, such as *Baieroxylon* Greguss, *Plaeoginkgoxylon* Feng, Wang *et* Roessler, *Primoginkgoxylon* Süß, *Proginkgoxylon* Zheng *et* Zhang and *Protoginkgoxylon* Zheng *et* Zhang *ex* Khudaiberdyev^5^,^8^,^9^,^10^,^11^,^12^,^13^. This profusion of works indicates the strong and lasting interest that the palaeobotany community has in *Ginkgo* wood evolution^5^,^14^,^15^.

These fossil records predating the Cretaceous are supposedly linked to the Ginkgoales; however, there is no evidence of a direct and certain systematic relationship with *Ginkgo*. Such relationships are hypothesized for isolated secondary xylem pieces, mostly on the basis of the occurrence of tracheid bunches, with their tips bent alongside the rays (intrusive tracheids), according to Greguss^8^. However, assigning Palaeozoic, Triassic or Jurassic woods to Ginkgoales represents a challenging task because there is no consensus with regards to Palaeozoic Ginkgophytes systematics or wood anatomy^16^, and the use of a single xylological feature (intrusive tracheids) is risky^17^, whereas several other features (e.g., mostly araucarian radial pitting) strongly depart from modern *Ginkgo* wood anatomy. To sum up, *Ginkgo* wood anatomy before the Early Cretaceous is still a matter of hypothesis.

Here, we report a new Middle to Late Jurassic fossil wood from northeastern China. While the leaf and reproductive structures of northeastern China-based *Ginkgo apodes* filled a 100 Myr gap in the fossil record of *Ginkgo* leaf and sexual organs^18^, the present fossil wood, at 153–165 Myr ago, is only slightly younger than *Ginkgo yimaensis* but represents the oldest known *Ginkgo* wood species^19^. It thus documents the earliest *Ginkgo* wood anatomy and establishes what could be called the missing “ancestral form” or the dawn for *Ginkgo* wood evolution.

In northeastern China, one of the major fossil wood localities is located in Changgao Town of Beipiao City, western Liaoning, including Lamaying, Shebudaigou, Taizishan, Toudaogou and Renjiagou villages. The fossil wood specimen was collected near Toudaogou village (121°00′–121°09′ E, 41°43′–41°47′ N) in Changgao Town of Beipiao City. The fossil wood locality and geological sections of the Tiaojishan Formation in Beipiao, western Liaoning, northeastern China are described by Wang *et al*.^20^.

Stratigraphically, the fossil wood specimen was preserved in the Tiaojishan Formation (previous known as the Lanqi Formation). This formation is widely distributed in western Liaoning Province and the neighbouring northern part of Hebei Province. The Tiaojishan Formation is usually considered to be Middle Jurassic in age, based on palaeontological assemblages^21^,^22^. Recent isotopic dating of 40Ar/39Ar in volcanic rock revealed, however, a transition between the late Middle Jurassic and the early Late Jurassic ages (approximately 153 Ma to 165 Ma) for the Tiaojishan Formation^23^,^24^.

The Tiaojishan Formation is 2000 m thick and is lithologically composed of intermediate lava and pyroclastic rocks, intercalated with basic volcanic rocks and sedimentary deposits^25^,^26^, with plant-bearing beds made of fine-grained sandstones intercalated with shales. These beds contain abundant well-preserved fossil plants, including leaf foliages, seeds and fruits, permineralized rhizomes and fossil wood^20^. Many anatomically preserved plant specimens were recently reported from the Tiaojishan Formation, including fern rhizomes *Ashicaulis*^27^,^28^,^29^,^30^, cycad stem *Lioxylon*^31^ and conifers *Araucariopitys, Pinoxylon, Sciadopityoxylon* and *Xenoxylon*^20^,^32^ (Fig. 1).

## Results

### Systematic palaeontology

 Class: Ginkgopsida

  Order: Ginkgoales

   Family: incertae sedis

    Genus: *Ginkgoxylon* Saporta

    Type species: *Ginkgoxylon gruetii* Pons *et* Vozenin-Serra

**Figure 2:**
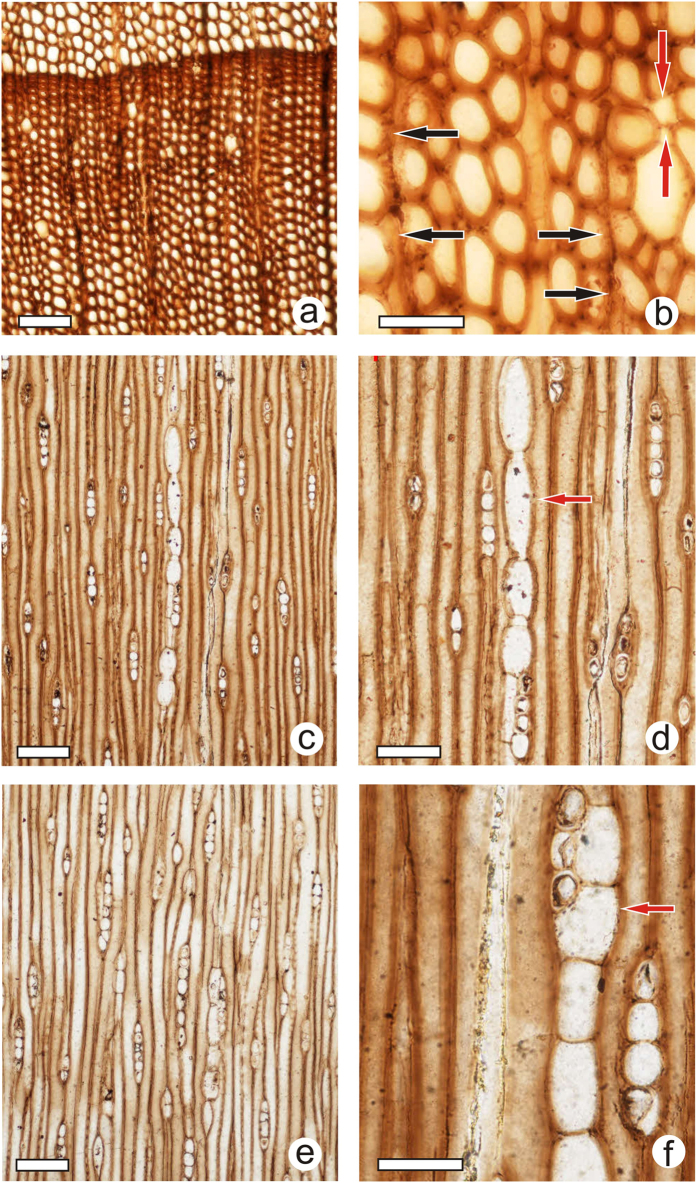
*Ginkgoxylon liaoningense* sp. nov. from western Liaoning, China. (**a**) Transverse section with a well-marked growth ring. Scale bar = 100 μm. (**b**) Transverse section with intercellular spaces (red arrows) and locally destroyed walls (black arrows), possibly resulting from a fungal attack. Scale bar = 40 μm. (**c**) Tangential section; note the tracheid tip contiguous to the ray margin. Scale bar = 100 μm. (**d**) Tangential section with inflated axial parenchyma associated to a ray (red arrow). Scale bar = 60 μm. (**e**) Tangential section with tracheid tips associated to inflated axial parenchyma or to ray parenchyma. Scale bar = 100 μm. (**f**) Tangential section with inflated axial parenchyma associated to a three-cell ray (red arrow). Scale bar = 40 μm.

**Figure 3:**
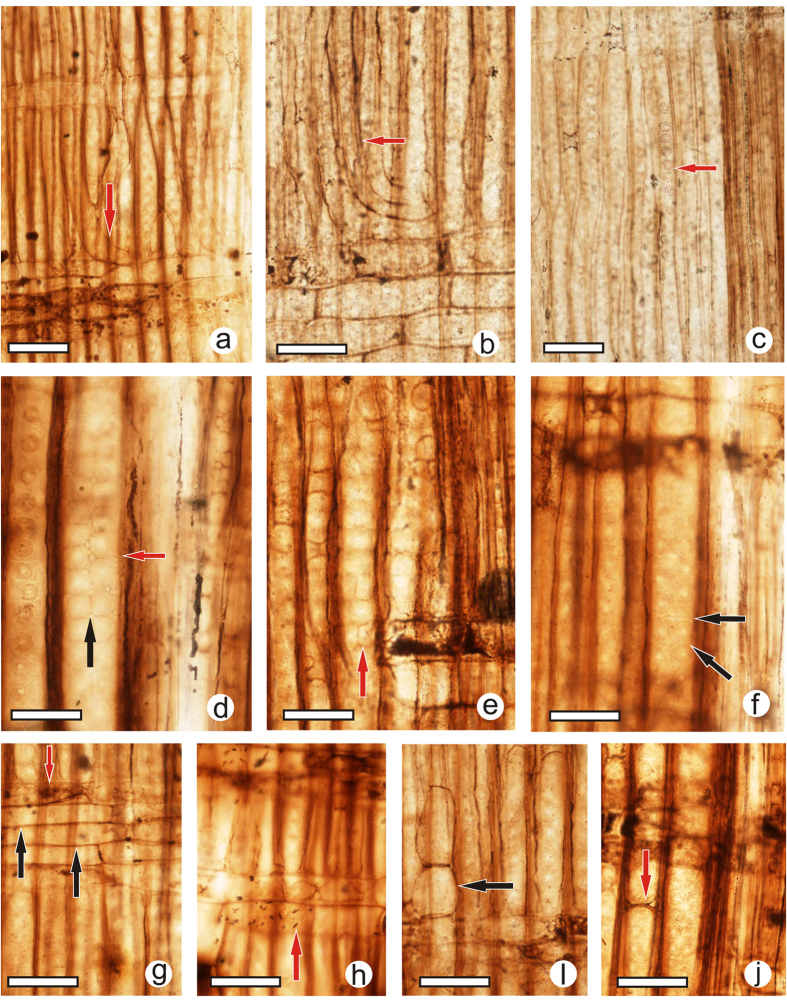
*Ginkgoxylon liaoningense* sp. nov. from western Liaoning, China. (**a**) A tracheid bunch (red arrow) with tips bent alongside wood rays. Scale bar = 80 μm. (**b**) A tracheid bunch (red arrow) with storied tips overlapping one another. Scale bar = 40 μm. (**c**) Tracheid radial pits (red arrow), mostly uniseriate, round and distant. Scale bar = 80 μm. (**d**) Tracheid radial pits contiguous and biseriate, either alternate (red arrow) or subopposite (black arrow). Scale bar = 40 μm. (**e**) Pairs of opposite tracheid radial pits within a uniseriate contiguous pit chain (red arrow). Scale bar = 40 μm. (**f**) Sanio’s rims (black arrows). Scale bar = 40 μm. (**g**) Ray cells, possible resiniferous content (red arrow) cells, and ray cell transverse walls (black arrows). Scale bar = 40 μm. (**h**) Cross-fields. Note that the cross-fields are (1) 2–4 cupressoid oculipores, with an oblique aperture (red arrow). Scale bar = 40 μm. (**i**) Axial parenchyma, with a possible crystalliferous chamber (black arrow). Scale bar = 40 μm. (**j**) A trabecula (red arrow). Scale bar = 40 μm.

*Ginkgoxylon liaoningense* sp. nov. Jiang, Wang, Philippe *et* Zhang

(Fig. 2a–f; Fig. 3a–j)

Type specimen: PB22285 and slides. (housed at the palaeobotanical collection of Nanjing Institute of Geology and Palaeontology, CAS).

Type locality: Toudaogou village, Beipiao City, Liaoning Province, China.

Type horizon: Tiaojishan Formation, Middle to Late Jurassic.

Etymology: after the fossil location in Liaoning Province.

Diagnosis: Tracheidoxyl with *Ginkgoxylon* features, including strongly inflated axial parenchyma chains, idioblasts, intrusive tracheid tips, ordered cupressoid oculipores in cross-fields, opposite pairs of radial pits separated by Sanio’s rims and an irregular aspect of the cross-section; similar to *G. chinense* Zhang, Zheng *et* Shang, but with approximately half the radial pits being contiguous and some imbricate biseriate alternate radial pits.

#### Description

The specimen PB22285 is preserved as a tracheidoxyl, with well-marked growth rings. The transition from early wood to late wood is gradual, with an intermediate type occupying most of the ring (Fig. 2a). The axial cells in cross-section are irregular in size and shape (Fig. 2b), and the tracheids are mostly quadrate to elliptical in the early wood, and more regularly narrowly rectangular in the late wood. Intercellular spaces are often distinct (Fig. 2b, red arrows), and the tracheid wall is thick, even in the early wood, suggesting a compression wood type. The tracheid walls are destroyed in some fungi infected areas (Fig. 2b, black arrows).

In a tangential section (Fig. 2c–f), xylem rays are homogeneous, relatively low, (1) 2–4 (15) cells high and are often associated with inflated axial parenchyma (Fig. 2d,f, red arrows). The tracheid tips are often contiguous to the ray margin or associated to inflated axial parenchyma (Fig. 2c,e).

In the radial section (Fig. 3a–j), some tracheid bunches are present, with storied tips bent alongside wood rays, and the tips sometimes overlap one another (Fig. 3a,b, red arrows). The tracheid radial pits are mostly uniseriate, round and distant (Fig. 3c, red arrow), sometimes contiguous and somewhat flattened, and locally biseriate. The pattern of biseriate pits is variable, from alternate crowded (Fig. 3d, red arrow) to sub-opposite crowded (Fig. 3d, black arrow) or opposite distant (Fig. 3e, red arrow), sometimes with Sanio’s rims (Fig. 3f, black arrows). Ray cells in each crossing field yield 4–6 tracheids in the early wood, and ray cell transverse walls are thin and unpitted (Fig. 3g, black arrows), often resiniferous (Fig. 3g, red arrow); cross-fields with (1) 2–4 cupressoid oculipores, with an oblique aperture (Fig. 3h, red arrow). Oculipores are ordered in columns and lines. The axial parenchyma are abundant and well distributed within the ring, with possible crystalliferous chambers (Fig. 3i, black arrow). Trabeculae are locally preserved (Fig. 3j, red arrow), whereas resin canals are not observed.

#### Comparison

Except for *Protoginkgoxylon*, most fossil wood genera with some *Ginkgo* type features are used for specimens with a strongly araucarian radial pitting. *Baieroxylon* Greguss has idioblasts but no inflated axial parenchyma chain. *Palaeoginkgoxylon* Feng, Wang *et* Roessler, based on a Permian type, has mixed pitting, but with opposite pairs only locally^5^. This is also the case for *Ginkgophytoxylon* Vozenin-Serra, Broutin *et* Toutin-Morin, which was erected based on a type from the Permian of France^33^. *Ginkgomyeloxylon* Giraud *et* Hankel, although diagnosed as having a “*Ginkgo* type of wood”, is based on a type with strongly araucarian radial pitting^15^,^34^. Similarly, *Ginkgoxylpropinquus* Savidge, despite its diagnosis, is based on a type with *Steinkerne*-preserved araucarian radial pitting^35^. This could probably also be the case for *Primoginkgoxylon* Süß, which has thick-walled idioblasts and uninflated axial parenchyma^12^.

*Protoginkgoxylon* was validly recognized by Zheng and Zhang^36^ based on Permian material (see supplementary note). The type material of *Protoginkgoxylon* Zheng *et* Zhang has idioblasts and axial parenchyma, but this is only slightly inflated, never reaching 3–4 tracheids in width as observed in our material. Its radial pitting is described as of the “protopinoid-type”, but the protologue illustrates a pitting much more araucarian than our material. It never shows opposite pit pairs separated by Sanio’s rim. Although they display some *Ginkgo*-like features, the other species included in *Protoginkgoxylon* Zheng *et* Zhang similarly differ from the material studied here by their mixed type of radial pitting being much more araucarian, with usually more than half of the pits being contiguous and/or alternate.

Anatomically, our new fossil wood material combines strongly inflated axial parenchyma chains (in addition to idioblasts), intrusive tracheid tips, cupressoid oculipores in its cross-fields, opposite pairs separated by Sanio’s rims and an irregular aspect of the cross-section. This combination of features is typical for *Ginkgo*^37^ and for *Ginkgoxylon* Saporta^11^. The diagnosis of *Ginkgoxylon* as emended by Khudaiberdyev^9^ does not preclude the inclusion of our material in this genus. Thus, although radial pitting includes a significant share of the araucarian type of radial pitting (approximately 40% to 50%), we assign the present fossil wood material to *Ginkgoxylon*. Within this genus, our material is most similar to the species of *Ginkgoxylon chinense* Zhang, Zheng *et* Shang from the late Early Cretaceous of Liaoning, but it is rather unique because of its partly araucarian radial pitting. Therefore, a new species is proposed for the present fossil wood material from the Jurassic of Beipiao, western Liaoning, China. A comparison of the xyological characters of fossil wood genera linked to Ginkopsida taxa is shown in Supplementary Table S1.

## Discussion

Ginkgoales has only one living species, *Ginkgo biloba*; however, this plant group has an extensive and diverse fossil record. This is evidenced by the numerous fossil leaf taxa, e.g., *Baiera* Braun, *Eretmophyllum* Thomas, *Ginkgoidium* Yokoyama, *Ginkgoites* Seward, *Ginkgoitocladus* Krassilov, *Glossophyllum* Kraeusel, *Pseudotorellia* Florin, and *Sphenobaiera* Florin^38^. Compared with these diverse leaf taxa, we still have a very limited knowledge of the history of Ginkgoales wood diversity. In contrast to most other gymnosperms and angiosperms, the wood of *Ginkgo* is particularly prone to degradation and hence, is less likely to get fossilized^39^. Although some fossil wood taxa have been documented based on Paleozoic material, which may be related to Ginkgoales, the fossil wood record of Ginkgoales will probably remain a matter of discussion for a long time.

Modern *Ginkgo* sometimes has a mixed type of radial pitting, especially in an area with disturbed growth. The amount of mixed radial pitting observed here is consequently not considered to preclude a relationship with *Ginkgo*. Because there is only one representative of the genus today, the past variability in *Ginkgo* wood anatomy was probably greater.

Wood with *Ginkgo biloba*-like anatomy is rare in the fossil record^6^,^40^. It is striking that the present fossil wood material in the Tiaojishan Formation, western Liaoning is found in association with several *Ginkgo* foliage species, including *G. huttonii, G. lepida* and G. *sibirica*^41^,^42^. In the same strata, however, *Ginkgoites* (with *G. tasiakouensis*) and *Sphenobaiera* (with *S. colchica* and *S. paucipartita*) also occur, and thus a link between the studied wood material and a particular foliage species cannot safely be hypothesized. Geologically, our fossil material is only slightly younger than *Ginkgo yimaensis. Ginkgo yimaensis* is probably the oldest *Ginkgo* with modern ovulate organs^18^ at ca. 170 Myr.

There are some other *Ginkgoxylon* species that have been described and putatively dated as Jurassic. *Ginkgoxylon quangnamense* Serra was reported based on material found *ex-situ* in Vietnam^43^. It might be Early Jurassic in age^44^; however, this generic identification must be confirmed because the type material was poorly preserved^43^. The case is similar with *Ginkgoxylon dixitii* Biradar *et* Mahabale from Andhra Pradesh of India^45^. This wood originates from the Kota Formation, which was long thought to be Early Jurassic in age; however, a recent palynological study demonstrated an Early Cretaceous age^46^. A *Ginkgoxylon* sp. was described from the Tendaguru strata in Tanzania^47^. This deposit is only partly Jurassic in age, and the fossil wood material originates from strata that possibly belong to the Aptian (Early Cretaceous)^47^.

With *Ginkgoxylon liaoningense* (Middle to Late Jurassic transition), we now have an ancestral form for a species series, continuing with *G. chinense* Zhang, Zheng *et* Shang (Aptian, Early Cretaceous), *G. gruettii* Pons *et* Vozenin-Serra (Cenomanian, Late Cretaceous), and *Ginkgo beckii* Scott, Barghoorn *et* Prakash (Miocene). Anatomical changes in this series are gradual and mostly limited to the radial pitting becoming more and more abietinean.

In conclusion, the fossil material described here from the Tiaojishan Formation in western Liaoning, China, dated as the Middle to Late Jurassic transition in age, is the oldest well-dated occurrence of the genus *Ginkgoxylon*. Although its anatomy departs slightly from that of the modern *Ginkgo*, it displays all its characteristic features. It differs only in having a more mixed type of radial pitting, which also occurs, albeit locally, in modern *Ginkgo* wood. The xylem structures of *Ginkgoxylon liaoningense* illustrates the basal state of *Ginkgo* wood anatomy and will contribute to the understanding of *Ginkgo* evolution.

## Methods

The fossil specimen used in this study was preserved as silicified wood. The techniques used for the investigation are the classical thin section method for silicified wood described in Jones and Rowe^48^. Nomenclatural and taxonomical positions follow those of Philippe^10^, Bamford and Philippe^49^ and Philippe and Bamford^11^. Photographs were taken with ACT-1C DXM1200C software adapted to a Nikon E600 transmitted light microscope. All fossil wood specimens and slides to which this study refers are housed in the Palaeobotany collection of the Nanjing Institute of Geology and Palaeontology, Chinese Academy of Sciences, in Nanjing (China), with registration numbers PB22285.

## Additional Information

**How to cite this article**: Jiang, Z. *et al*. A Jurassic wood providing insights into the earliest step in *Ginkgo* wood evolution. *Sci. Rep.* **6**, 38191; doi: 10.1038/srep38191 (2016).

**Publisher's note:** Springer Nature remains neutral with regard to jurisdictional claims in published maps and institutional affiliations.

## Supplementary Material

Supplementary Note

Supplementary Table S1

## Figures and Tables

**Figure 1:**
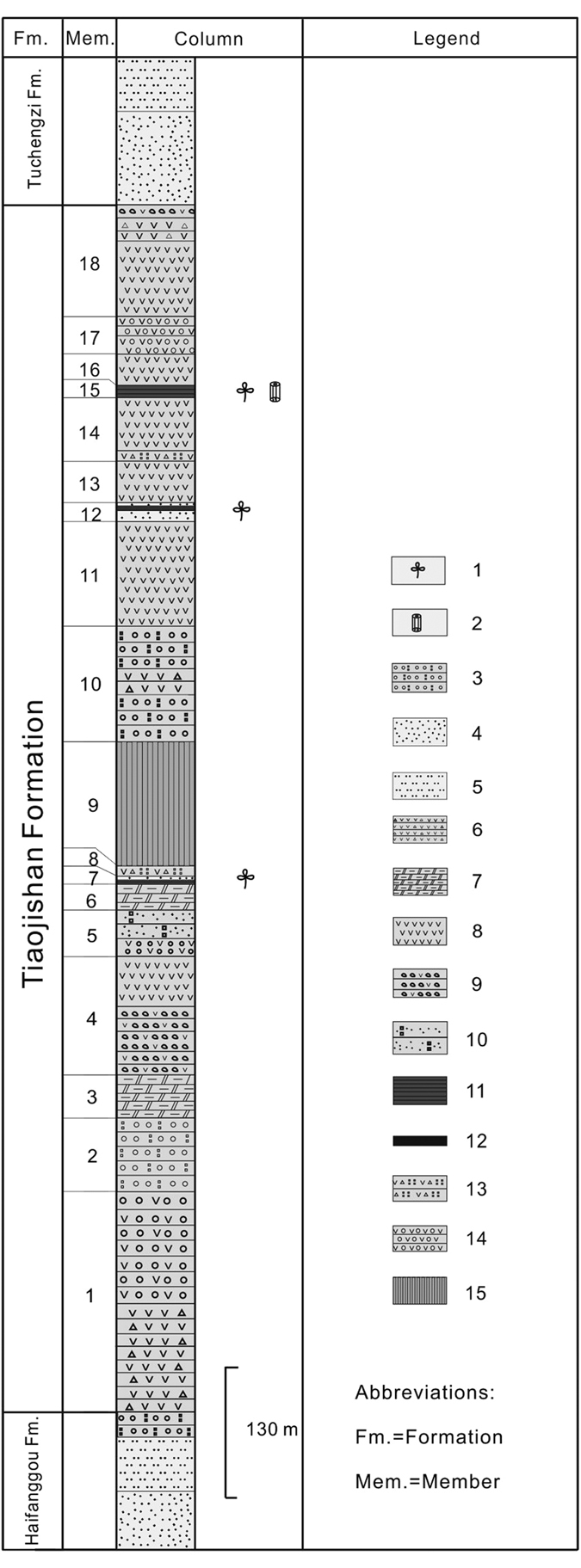
Stratigraphic column of the Tiaojishan Formation in Beipiao, Liaoning Province, China (Drawing based on lithological descriptions of Wang *et al*.^22^). 1. Plant fossils; 2. Fossil wood; 3. Andesitic conglomerate; 4. Sandstone; 5. Siltstone; 6. Andesitic brecciated lava; 7. Andesitic lava breccias; 8. Andesite; 9. Andesite agglomerate; 10. Conglomeratic tuffaceous sandstone; 11. Shale; 12. Coal seam; 13. Andesitic brecciated tuff; 14. Tuffaceous conglomerate; 15. Quaternary sediments.
