# Supplementary material for: A Jurassic wood providing insights into the earliest step in Ginkgo wood evolution
Source: Sci Rep. 2016 Dec 16;6:38191. doi: 10.1038/srep38191 (PMC5159789; doi:10.1038/srep38191)
Supplement: Supplementary Note [file srep38191-s1.pdf]

Supplementary Note

Supplementary Note 1: Nomenclatural notes on the fossil wood genus *Protoginkgoxylon*

A Jurassic wood providing insights into the earliest step in *Ginkgo* wood evolution

Zikun Jiang, Yongdong Wang*, Marc Philippe, Wu Zhang, Ning Tian, Shaolin Zheng

The genus *Protoginkgoxylon* has an intricate history, which is only partially reported by Zheng and Zhang[1]. It was first described by Khudaiberdyev in an unpublished thesis before published in 1971[2]. It is thus not validly published in this work, furthermore, two species being included without designation of a type (ICBN, art. 37.1). Moreover, the protologue includes two names which are both new combinations: *P. dockumense*, based on *Voltzioxylon dockumense* Torrey, which is the type of *Voltzioxylon*[3, 4]; and *P. catenatum*, based on *Protocupressinoxylon catenatum* Schultze-Motel, which was validly published only much latter[5]. Zheng and Zhang[1] advocated the use of *Protoginkgoxylon* and gave a diagnosis; they selected a type species, *P. benxiense*, based on material from the Early Permian of China. Later, Zheng and Zhang[6] considered that the name *Protoginkgoxylon* was invalidly published by Khudaiberdyev, and thus proposed the new genus name *Proginkgoxylon* Zheng *et* Zhang, transferring *Protoginkgoxylon benxiense* to this new genus[6]. However, it should be noted that the ICBN does not forbid the use of previously invalidly published names, although it might be a source of confusions. Therefore, the genus name *Protoginkgoxylon* reported by Zheng & Zhang[1] is validly published, including its type species. The genus *Proginkgoxylon* erected by Zheng *et* al.[6] may represent an illegitimate junior synonym of *Protoginkgoxylon* Zheng *et* Zhang.

References:

1. Zheng, S. L. & Zhang, W., Late Paleozoic ginkgoalean woods from northern China. *Acta Palaeontologica Sinica* **39**, 119–126 Supl (2000).

2. Khudaiberdyev, R., Data on Ginkgoales fossil wood (in Russian). In Sixtel, T. A., Kuzichkina, Y. M., Savitskaya, L. I., Khudaiberdyev, R., Shetsova, E. M. (eds.) History of Gingkoales evolution in Middle Asia. *Palaebotanica Uzbekistana* **2**, 98-104 (1971).

3. Torrey, R. E., The comparative anatomy and phylogeny of the Coniferales. Part 3. Mesozoic and Tertiary Coniferous Woods. *Memoirs of the Boston Society of Natural History* **6** (2),39–106 (1923).

4. Philippe, M., Nomenclature générique des trachéidoxyles mésozoïques à champs araucarioïdes. *Taxon* **42**, 74-80 (1993).

5. Müller-Stoll, W. R. &Schultze-Motel, J., Gymnospermen-Hölzer des deutschen Jura.Teil 2: die protopinoiden Hölzer. *Zeitschrifft der deutschengeologischen Gesellschaft* **140**, 53-71 (1989).

6. Zheng, S. L. et al., *Fossil woods of China* (ed. Zheng, S.L. et al.).1-356 (China Forestry Publishing House, 2008).
