# Supplementary material for: A Jurassic wood providing insights into the earliest step in Ginkgo wood evolution
Source: Sci Rep. 2016 Dec 16;6:38191. doi: 10.1038/srep38191 (PMC5159789; doi:10.1038/srep38191)
Supplement: Supplementary Table S1 [file srep38191-s2.pdf]

Supplementary Table S1

Supplementary Table S1: Comparison of xyological characters of fossil wood genera linked to Ginkopsida taxa

A Jurassic wood providing insights into the earliest step in *Ginkgo* wood evolution

Zikun Jiang, Yongdong Wang*, Marc Philippe, Wu Zhang, Ning Tian, Shaolin Zheng

Comparison of xyological characters of fossil wood genera linked to Ginkgopsida taxa

Genera	Characters (type of pitting; pith; Primary xylem)	Locality	Horizon; age	References
*Ginkgoxylon*Saporta	Inflated axial parenchyma chains (not only idioblasts), intrusive tracheid tips, cupressoidoculipores in its cross-fields, opposite pairs separated by Sanio's rims and an irregular aspect of the cross-section.	Beipiao, Liaoning, China	Tiaojishan Formation, Jurassic;<br>Shahai Formation, Early Cretaceous	Philippe &Bamford, 2008[1]
*Baieroxylon*Greguss	Idioblasts with no inflated axial parenchyma; secondary xylem homoxylic without secretory channels. Growth-rings very thick with narrow late wood. Tracheid section polygonal to rounded, variable in size. Radial tracheid pits areolate, circular, not covering the whole radial wall, mostly uniseriate, spaced to contiguous, occasionally biseriate alternate. In some areas tracheid ends recurved alongside the rays. Wood rays homogeneous, uniseriate, some being locally biseriate. Six rays by tangential millimeter, with rays by square millimeter in density. Cross-fields with 1-2 cupressoid pitting. Axial wood parenchyma occurs.	South America;<br>South RewaGondwana Basin, Madhya Pradesh, India;	Upper Triassic; Jurassic	Greguss, 1961[2]
*Primoginkgoxylon*Süß, Rōssler, Boppré & Fischer	Wood composed of tracheids, crystalidioblasts and rays, growth layers present, in the cross section tracheids in radial rows, crystals in tracheids presents, bordered pits on radial tracheid walls arranged abietoid, crystalidioblasts with circular diameter scattered over the crosses section; cross-field pits cupressoide; thick walled idioblasts with uninflated axial parenchyma.	Mombasa Basin, Kenya	Mazeras Formation<br>the Late Triassic	Süß et al., 2009[3]
Protogingkoxylon<br>Zheng& Zhang	Pith is solid, circular; 20–30 mm in diameter. Pitted cells irregularly distributed in the parenchymatous pith. Primary xylem is endarch, PX to MX, spiral, annular and scalariform/reticulate thickenings. araucarian radial pitting; opposite pairs only locally e.g. Sanio's rim.	Hulstai, Inner Mongolia;<br>Liaoning, China	Lower Shihhotse Formation;<br>Guadalupian, Permian;	Zheng& Zhang, 2000[4]<br>Zheng et al., 2008[5]

*Ginkgophytoxylon*Vozenin-Serra	idioblasts but no inflated axial parenchyma	France	Permian	Vozenin-Serra et al., 1991[6]
*Ginkgomyeloxylon*Giraud et Hankel	With pith, primary xylem and secondary xylem features; Ginkgo type of wood with araucarian radial pitting	Argentina	Middle Jurassic	Giraud and Hankel, 1986[7]; Gnaedlinger, 2012[8]
Ginkgoxylpropinquus Savidge	Pycnoxylic secondary xylem; in cross section, oft-disorganized tracheid radial files, tracheids of varied tangential diameter, axial parenchyma; in radial section, ray cells long, swollen, uniformly thin-walled with ergastic contents, cross-field pits (1-3) cupressoid with openings inclined c. 60o, long uniseriate chains of abietinoid bordered pits separated by weak crassulae, occasionally biseriate chains of non-araucoid pits arranged alternately sometimes oppositely; in tangential section, high frequency uniseriate rays to 16 cells in height, single-celled rays common, 1-2 biseriate cells rarely within uniseriate rays, cells upright of variable width.	Arizona, USA	Late Triassic	Savidge, 2006[9]

References

1.Philippe, M. & Bamford, M., A key to morphogenera used for Mesozoic conifer-like woods. *Review of Palaeobotany and Palynology* **148**, 184-207 (2008).

2. Greguss, P., Permische fossile Hölzer aus Ungarn. *Palaeontogr. Abt. B* **109**, 131–146 (1961).

3. Süß, H., Rößler, R., Boppré, M. &Fischer, O. W., Drei neue fossile Hölzer der Morphogattung *Primoginkgoxylon* gen. nov. aus der Trias von Kenia. *Feddes Repertorium***120** (5/6), 273-292 (2009).

4. Zheng, S. L.& Zhang, W., Late Paleozoic ginkgoalean woods from northern China. *Acta Palaeontologica Sinica* **39**, 119–126 Supl (2000).

5. Zheng, S. L. et al., *Fossil woods of China* (ed. Zheng, S.L. et al.). 1-356 (China Forestry Publishing House, 2008).

6. Vozenin-Serra, C., Broutin, J. & Toutin-Morin, N., Bois permiens du sud-ouest de l’Espagne et du sud-est de la France. Implications pour la taxonomie des gymnospermes paléozoïques et la phylogénie des Ginkgophytes. *Palaeontographica Abteilung B* **221**, 1-26 (1991).

7. Giraud, B. &Haenkel, O., Nouveaux bois fossiles des dépôts du Karoo du Bassin de Luwegu (Tanzanie méridionale). *Annales de Paléontologie* **72**(1), 1-27 (1986).

8. Gnaedinger, S., Ginkgoalean woods from the Jurassic of Argentina: taxonomic considerations and palaeogeographical distribution. *Geobios* **45**, 187-198 (2012).

9. Savidge, R. A., Xylotomic evidence for two new conifers and a *Ginkgo* within the Late Triassic Chinle Formation of Petrified Forest National Park,

Arizona, USA. In: Parker, W. G., Ash, S. R., Irmis, R. B. (Eds.), A Century of Research at Petrified Forest National Park: Geology and Paleontology. *Museum of Northern Arizona special publications* **62**, 147–149 (2006).
